# Biochar-Enhanced Resistance to *Botrytis cinerea* in Strawberry Fruits (But Not Leaves) Is Associated With Changes in the Rhizosphere Microbiome

**DOI:** 10.3389/fpls.2021.700479

**Published:** 2021-08-23

**Authors:** Caroline De Tender, Bart Vandecasteele, Bruno Verstraeten, Sarah Ommeslag, Tina Kyndt, Jane Debode

**Affiliations:** ^1^Plant Sciences Unit, Flanders Research Institute for Agriculture, Fisheries and Food (ILVO), Merelbeke, Belgium; ^2^Department of Applied Mathematics, Computer Science and Statistics, Ghent University, Ghent, Belgium; ^3^Epigenetics and Defence Research Group, Department Biotechnology, Ghent University, Ghent, Belgium

**Keywords:** biochar, microbiome, strawberry, plant defense, metabarcoding, RNA sequencing

## Abstract

Biochar has been reported to play a positive role in disease suppression against airborne pathogens in plants. The mechanisms behind this positive trait are not well-understood. In this study, we hypothesized that the attraction of plant growth-promoting rhizobacteria (PGPR) or fungi (PGPF) underlies the mechanism of biochar in plant protection. The attraction of PGPR and PGPF may either activate the innate immune system of plants or help the plants with nutrient uptake. We studied the effect of biochar in peat substrate (PS) on the susceptibility of strawberry, both on leaves and fruits, against the airborne fungal pathogen *Botrytis cinerea*. Biochar had a positive impact on the resistance of strawberry fruits but not the plant leaves. On leaves, the infection was more severe compared with plants without biochar in the PS. The different effects on fruits and plant leaves may indicate a trade-off between plant parts. Future studies should focus on monitoring gene expression and metabolites of strawberry fruits to investigate this potential trade-off effect. A change in the microbial community in the rhizosphere was also observed, with increased fungal diversity and higher abundances of amplicon sequence variants classified into *Granulicella*, *Mucilaginibacter*, and *Byssochlamys* surrounding the plant root, where the latter two were reported as biocontrol agents. The change in the microbial community was not correlated with a change in nutrient uptake by the plant in either the leaves or the fruits. A decrease in the defense gene expression in the leaves was observed. In conclusion, the decreased infection of *B. cinerea* in strawberry fruits mediated by the addition of biochar in the PS is most likely regulated by the changes in the microbial community.

## Introduction

Biochar, a by-product of pyrolysis, is the subject of many agricultural studies (Lehmann and Joseph, 2009). Particularly for soilless cultivation, biochar has been reported to be beneficial: first, because it can partially replace peat (Blok et al., 2017), and second, because it improves plant-based traits (De Tender et al., 2016b; Nieto et al., 2016). Biochar application improves structural stability and water- and air-holding capacity. Biochar can successfully replace up to 25% of bulk peat (Lehmann and Joseph, 2009; Blok et al., 2017; Dumroese et al., 2018; Chrysargyris et al., 2019). However, even in small quantities, biochar has beneficial effects on agricultural and horticultural crops. For instance, the addition of 2 g/L of biochar to white peat enhances root formation, plant biomass, and fruit production in strawberry plants (De Tender et al., 2016a, b). In addition, low concentrations of biochar in growing media have been reported to support disease suppression (Frenkel et al., 2017). Symptoms of airborne diseases caused by pathogens, such as *Botrytis cinerea*, *Colletotrichum acutatum*, *Podosphaera aphanis*, and *Leveillula taurica* (Elad et al., 2010; Meller Harel et al., 2012; De Tender et al., 2016a, b), as well as soilborne pathogens, such as *Pythium*, parasitic nematodes, and *Fusarium oxysporum* (Huang et al., 2015; Frenkel et al., 2017; Jaiswal et al., 2018, 2020), are reduced on the application of biochar to the growing media or soil. No direct toxic effect of biochar on pathogens has been reported (e.g., Huang et al., 2015; Wang et al., 2020), indicating that other mechanisms play a role in disease suppression.

Nutrient availability for the host plant is an important factor in disease control (Agrios, 2005). Previous studies have shown that biochar can act as fertilizer and thus influences the nutrient availability in the growing medium, leading to increased phosphorus (P) contents in wheat and strawberry (Olmo et al., 2016; Amery et al., 2021), potassium (K) concentration in strawberry (Amery et al., 2021), magnesium (Mg) and manganese (Mn) contents in maize plants (Glaser et al., 2015), and nitrogen (N) and P levels and uptake efficiency in tomato and sweet pepper (Levesque et al., 2020). Nutrient deficiencies and toxicities can directly affect plant disease resistance and tolerance (Marschner, 1995; Agrios, 2005). K for instance can decrease the susceptibility of the plant up to the optimal level for growth (Huber and Graham, 1999). Another important nutrient in terms of disease resistance is calcium (Ca), which is important for the stability and function of plant membranes and an important component of the cell wall. Ca also plays a role in pattern-triggered immunity (PTI) responses, where Ca^2+^ bursts lead to activation of Ca-dependent protein kinase signaling (Bredow and Monaghan, 2018). A drop in the Ca level in the plant can therefore result in an increased susceptibility to pathogens (Dordas, 2008). However, the extensive use of nutrients can also be harmful to the plants. For instance, excessive N concentration can lead to increased susceptibility to plant pathogens and higher rates of infection (Nam et al., 2006; Dordas, 2008; De Tender et al., 2021).

The positive impact of biochar on plant health has been linked to the changes in the microbial community of the rhizosphere of the plant—the zone of soil or growing medium located immediately around the plant root (Philippot et al., 2013; De Tender et al., 2016b; Jaiswal et al., 2017; Kolton et al., 2017). The addition of biochar can change the microbial community (Palansooriya et al., 2019; Wu et al., 2020) of the rhizosphere, with more abundance of the above-described plant growth-promoting rhizobacteria (PGPR) and fungi (PGPF), such as members of the genera *Pseudomonas*, *Bacillus*, and *Trichoderma* (Bakker et al., 2013; Mehari et al., 2015; De Tender et al., 2016a, b; Jaiswal et al., 2018). However, the longevity of the microbial changes in the rhizosphere is unknown and requires investigation (Jaiswal et al., 2019).

The microbial mechanisms associated with disease suppression in plants include the competition for nutrients, antibiosis, and induced resistance (IR) in host plants (Bakker et al., 2013). For foliar pathogens specifically, the latter is proposed to be the main mode of action, as root colonization by PGPR and PGPF can promote IR (Bakker et al., 2013; Mehari et al., 2015; Samain et al., 2017, 2019). Inducing the innate defense system of plants through the application of biochar can reduce the susceptibility to diseases by activating the “Induced resistance” phenotype. This phenotype occurs in plants after they are triggered by certain pathogens, pests, beneficial microbes, chemical agents, physical wounding, or herbivory, where they exhibit enhanced resistance against future challenges when compared with naïve control plants (De Kesel et al., 2021). Two major forms of IR have been described in plants, namely, induced systemic resistance (ISR) and systemic acquired resistance (SAR), where ISR is most commonly attributed to the presence of PGPR and PGPF and is often related to phytohormones like ethylene and jasmonic acid (Pieterse et al., 2014), and SAR can be triggered by both chemical and biological elicitors and is mainly mediated through the phytohormone salicylic acid (Sticher et al., 1997; Vlot et al., 2009). The establishment of IR is not always correlated with a strong direct defense response on the application of an IR trigger. In some cases, IR is associated with earlier, stronger, and/or faster activation of defense on pathogen attack when compared with non-IR plants, a phenomenon known as “(defense) priming” (De Kesel et al., 2021). Induction of ISR *via* biochar application has been shown for airborne pathogens (Elad et al., 2010; Meller Harel et al., 2012; Mehari et al., 2015) and soilborne pathogens (Huang et al., 2015; Jaiswal et al., 2020).

Previously, we have shown that amending PS with biochar made from holm oak feedstock leads to a reduced susceptibility to *B. cinerea* in strawberry (De Tender et al., 2016a, b). From the study by De Tender et al. (2016a), we hypothesized that this increased disease resistance might be related to either one or a combination of three mechanisms.

The first mechanism involves a change in the nutrient composition in the plant leaves, with higher uptakes of nutrients, such as K, N, and Ca. In previous study, we showed that the addition of biochar results in higher nutrient concentrations in the PS (De Tender et al., 2016a, b), and we did not know so far if this also leads to a higher uptake of nutrients by the plants and increased concentrations in the plant leaves and fruits. Therefore, in this study, the nutritional status of both the growing medium and the strawberry plant leaves and fruits are measured.

The second mechanism is an induction of the immune system or defense priming of plants. In this study, we measured the expression of plant defense genes by using reverse transcription quantitative PCR (RT-qPCR) and RNAseq at several time points after inoculation of strawberry with *B. cinerea*.

The final mechanism is the attraction of PGPR and PGPF to the plant root. From a previous study, we knew that biochar has the potential to alter the rhizosphere microbial community as measured using 16S and ITS2 rRNA gene metabarcoding. In this study, we also analyzed the microbiome of the bulk peat substrate (BS) to verify that these changes in the rhizosphere are due to an active attraction or stimulation of PGPR in the zone in close collaboration with the plant root. Thus, we demonstrated a “rhizosphere” effect of the biochar rather than a “generalized” biochar effect.

## Materials and Methods

### Experimental Design

Strawberry plants (*Fragaria × ananassa* cultivar Elsanta) were grown in 1.5 L pots containing NOVOBALT white peat 100% (AVEVE Lammens, Wetteren, Belgium), mixed with 0.35 g/L of Ecomix organic fertilizer, 1.43 g/L of lime (RHP, MG’s-Gravenzande, The Netherlands), and 0.35 g/L of Haifa Multi-mix 14 + 16 + 18 (+micronutrients) PGMix fertilizer (Haifa North-West Europe). Half of the PS mixture was used without amendments (PS), while the other half was mixed with 2 g of dry matter (DM) biochar/L PS (PS + BC). Biochar was prepared from holm oak at 650°C for 12–18 h (as used in previous studies; De Tender et al., 2016a, b). To obtain 40% water-filled pore space (WFPS), all pots were wetted and put in a closed bag to pre-incubate at 15°C for 1 week. During plant growth, no additional fertilizer was used. Based on the loss of mass, the moisture content of the substrate was adjusted each week to 40% WFPS by adding water to the tray under the pot. Cold-stored bare-root strawberry (*Fragaria* × *ananassa*, cultivar Elsanta) transplants were planted in half of the pots; the other half contained one of the BS mixtures (PS or PS + BC). Pots with and without plants were arranged in the greenhouse in a semi-randomized design as described by De Tender et al. (2021) and grown at 20°C for 11 weeks. In total, six biological replicates were sampled every week per time point × growing medium combination. At week 8 of plant growth, the *B. cinerea* bioassay was performed on the leaves of the remaining plants.

### Measurement of Plant Physiological Parameters

Strawberry plants were sampled every week. The aboveground biomass [fresh weight (FW) and dry weight (DW, 48 h at 70°C)] and the belowground root system were weighed after sampling for the rhizosphere microbiome analysis and rinsing the roots with sterilized water (see section “Microbial analysis”).

From week 6 onward, fruits started to appear on the plants. Per plant and at four time points (week 6 until 9), ripened fruits were picked, counted, and weighed (FW). A part of these fruits was inoculated with *B. cinerea* after picking (see section *“Botrytis cinerea* bioassay”). The remaining fruits were used for chemical characterization.

### Chemical Characterization of the PS, Plant Leaves, and Strawberry Fruits

The PS was sampled every week during the entire duration of the experiment (11 weeks). In total, three biological replicates per condition were studied. The DM content was determined according to EN 13040. Electrical conductivity (EC; EN 13038) and pH–H_2_O (EN 13037) were measured in a 1:5 soil to water (v/v) suspension. Water-extractable PO_4_-P, NO_3_-N, Cl, SO_4_, NH_4_-N, C, Cu, Si, K, Ca, Mg, and Na concentrations were measured with either a 5110 VDV Agilent ICP-OES, Dionex DX-3000 IC ion chromatograph or a Skalar SAN++ flow analyzer, as described by De Tender et al. (2019).

In addition, plant leaves of three biological replicates [per time point × mixture (PS, PS + BC)] were chemically analyzed at weeks 3, 6, and 9 of the experiment. Leaves were dried at 70°C and ground, and the material of the two plants was mixed and considered as one biological replicate. Fruits were freeze-dried before grinding. Total N was determined by using the Thermo Scientific—flash 4000 N analyzer (ISO 16634-1), and the total concentrations of P, K, Mg, and Ca were determined by using 5110 VDV Agilent ICP-OES in the extract based on adding 10 ml of HCl (1N) to the ash after incineration of 0.5 g for minimum 6 h at 450°C. The assessment of the optimal range and nutrient deficiency for foliar composition was based on the study by Pritts et al. (2015). The total uptake in the leaves and fruits was calculated by multiplying the measured concentration by the dry mass of leaves/fruits.

### Microbial Analysis

The analysis of the bacterial and fungal microbial community was done each week for six biological replicates taken from either the strawberry rhizosphere (pots with plants), according to Lundberg et al. (2012), or the BS (pots without plants) by sampling 250 mg of medium per pot. The DNA of the samples was extracted using the DNeasy PowerSoil Kit (Qiagen, Hilden, Germany) according to the instructions of the manufacturer and stored at −20°C before use.

Metabarcoding of the bacterial and fungal rhizosphere was done on the V3–V4 fragment of the 16S rRNA gene and the ITS2 gene fragment, respectively (Illumina, San Diego, CA, United States). Library preparation, quality control, and pooling were done as described by De Tender et al. (2016b). The resulting libraries were sequenced using Illumina MiSeq v3 technology (2 × 300 bp) by Admera Health, South-Plainfield, NJ, United States, spiked with 30% PhiX DNA.

Demultiplexing of the raw sequencing reads was done by the sequencing provider. Reads are available for download at the NCBI sequence read archive (SRA) under project numbers PRJNA576171 and PRJNA576339 for the bacterial and fungal sequences, respectively.

Trimming, filtering, merging of the reads, dereplication, sorting, amplicon sequence variant (ASV) calling, and chimera removal were done using the DADA2 algorithm v1.10 (Callahan et al., 2016) and described in detail by De Tender et al. (2021).

### *Botrytis cinerea* Bioassay

After 8 weeks of plant growth, leaves of half of the plants were inoculated with *B. cinerea* (strain PCF895; Debode et al., 2013) according to the method of Meller Harel et al. (2012), described in detail by De Tender et al. (2016b). The other half of the plants were inoculated with sterile potato dextrose agar plugs. The resulting lesions were recorded 1 week after inoculation using a 0–4 disease scale (De Tender et al., 2021). This scoring was used to calculate the disease severity index (DSI) per plant (*i*), used as input for statistical analysis.

$${\text{DSI}}_{i}=100\times \frac{1\times n_{i1}+2\times n_{i2}+3\times n_{i3}+4\times n_{i4}}{4\times n_{i}}$$

where *n*_*i*1_,…,*n*_*i*4_ represent the number of leaves of each infection score, and $n_{i}=\sum_{l=0}^{4}n_{il}=9$ is the number of leaves measured for each plant. This index has values in the interval [0, 100], with a minimum index if all leaves score 0 and a maximum if all leaves score 4. Inoculated leaves remained on the plant until the end of the experiment.

From the time that fully ripened strawberry fruits appeared, they were picked, and 10 fruits per treatment were inoculated with *B. cinerea* according to the method of Bhaskara Reddy et al. (2000) and described in detail by De Tender et al. (2016b). The area under the disease progress curve (AUDPC) was calculated based on the disease scores (Campbell and Madden, 1990; Schandry, 2017). In total, the inoculation was repeated independently four times for both experiments.

### RNA Extraction and Gene Expression Analysis

Six plugs (ø 0.5 cm) of plant leaves were collected per plant, pooled, flash-frozen in liquid nitrogen, and stored at −80°C until use. Samples were taken from plant leaves 1 week after inoculation (qPCR and RNAseq), from inoculated and non-inoculated plants. To avoid excessive necrosis, leaves with an infection score of 1 were taken from the plants inoculated with *B. cinerea* (Windram et al., 2012).

Gene expression was analyzed in leaves from strawberry plants grown in (1) PS, mock-inoculated (PS), (2) biochar-enriched PS, mock-inoculated (PS + BC), (3) PS and *B. cinerea* inoculated (PS + I), or (4) biochar-enriched PS and *B. cinerea* inoculated (PS + BC + I). Three biological replicates were taken for each sample type. RNA was extracted from the plant leaves according to Luypaert et al. (2017) and described in detail by Debode et al. (2018). DNase treatment was done using the DNA-free Kit (Ambion, Thermo Fisher Scientific). For cDNA synthesis, the Tetro cDNA Synthesis Kit (Bioline, Meridian Bioscience, London, United Kingdom) was used, starting from 1.5 μg of DNA-free RNA.

First, the gene expression was studied by using RT-qPCR on nine genes (three reference genes and six defense-related genes), as described in detail by Debode et al. (2018). We used SYBR Green as implemented in the SensiMix-SYBR-no-ROX-Kit (Meridian Bioscience, London, United Kingdom) of GC biotech for RT-qPCR.

Second, to obtain a comprehensive overview, RNA sequencing was used to study the overall gene expression of the plant leaves. Library construction, sequencing, read filtering, and trimming were done as described in detail by De Tender et al. (2021). Raw reads can be found in the Gene Expression Omnibus data accession number GSE144526. The resulting reads were mapped to the *F. ananassa* reference genome (Hirakawa et al., 2014) with STAR v2.6.1d (Dobin et al., 2013). Based on the resulting alignment files, a count table was built (R package GenomicAlignments; Lawrence et al., 2013), taking only uniquely mapping reads into account. This count table was further used for differential expression analysis with edgeR.

RNA sequencing results were analyzed in more detail using the Gene Ontology (GO) enrichment analysis. This tool uses a distinct set of gene IDs based on different *Fragaria vesca* annotations. To convert the gene IDs of the *F. ananassa* reference genome, the annotation and corresponding gene sequences were retrieved for *F. vesca* through the *F. vesca* v1.1 assembly from Phytozome (version 13; Goodstein et al., 2012). Corresponding gene sequences were used to create a BLAST database using a locally installed version of the NCBI BLAST service (v2.9.0+; Tao, 2008). The database was subsequently masked using the DustMasker algorithm. Gene sequences of *F. ananassa* were blasted against each of these databases. GO visualization was done using REVIGO (Supek et al., 2011). AgriGO was used for the parametric analysis of gene set enrichment using the log2 fold change values of all genes. Benjamini and Hochberg false discovery rate (FDR) correction was performed using the default parameters to adjust the *p*-value. Genes were considered to be differentially expressed at FDR <0.05. Two-sided binomial tests were used to determine the significant tendencies toward upregulation or downregulation.

### Statistical Analysis

Chemical concentrations of the PS mixtures (PS, PS + BC), plant leaves and fruits, and the diversity measures from the bacterial and fungal community were statistically analyzed with a general linear model (LM) in which biochar treatment and time were set as two factors. To obtain data normality, the concentration of nutrients was log-transformed. Homogeneity of variances was checked using box plots.

The temporal evolution of the FW, DW, and root FW content of plants was expected to be nonlinear. Therefore, a generalized additive model was used to analyze these response variables, making use of a loess smoother, as described by De Tender et al. (2021).

Before starting the analysis of shifts in the bacterial and fungal community, the ASV tables were first filtered to remove low abundant counts. ASVs with a count of two in at least six samples were kept for the analysis. The multivariate analysis was done using the R package vegan (version 2.5.4) (Oksanen et al., 2010). A dissimilarity matrix was built, based on the Bray–Curtis dissimilarity index, from the ASV table as generated by DADA2 for both bacterial and fungal sequences. Homogeneity of variances was checked on this dissimilarity matrix by using the betadisper function. Furthermore, the significance of biochar treatment, time, sampling type (BS or rhizosphere), and the interaction factors were analyzed using the PERMANOVA analysis in which the Bray–Curtis dissimilarity matrix was used as an input and visualized by a principal coordinate analysis (PCoA) plot. In addition, differential abundances were assessed using likelihood-ratio tests, for which we tested the effect of biochar treatment within each time point, specifically for the rhizosphere samples. The analyses were done at the ASV level, but visualizations were made by clustering the samples at the genus level. These analyses were done using an edgeR package, version 3.24.3 (Robinson et al., 2010), and were described in more detail by De Tender et al. (2016a).

For plant leaf infection, the DSI was used as a response variable for the disease score. A linear mixed effect model was fitted with days after inoculation (DAI) (7, 9, and 12 days) and biochar treatment as fixed main effects and the plant as the random effect. For *B. cinerea* infection on fruits, the AUDPC value was used as the disease index. According to the study by Schandry (2017), a generalized LM was used with biochar treatment and repeated (infection was scored at four independent time points) as main effects.

To analyze the differential gene expression (DE) based on the RT-qPCR data, the relative quantification technique was used and described in detail by Debode et al. (2018). Statistical significance of the DE levels was analyzed using an LM in which biochar treatment and *B. cinerea* infection were set as main effects and the interaction treatment × infection was taken into account. DE genes were analyzed using edgeR version 3.4.3 for the RNA sequencing data. After a trimmed mean of *M* value normalization, a negative binomial model with main effects for treatment and infection as well as treatment × infection interaction was used. Significant DE genes were obtained by using the likelihood-ratio tests on the appropriate contrasts.

## Results

The aim of this study was to investigate if and how biochar amendment can protect strawberry from infection by the fungal pathogen *B. cinerea* on leaves and fruits. We postulated that the changes in the microbiome are essential for the effect of biochar amendment on plant disease resistance and that microbial changes in the rhizosphere lead to (1) a change in the nutrient content of the plant and/or (2) activation and/or priming of plant defense genes. First, we evaluated the effect of biochar on the disease resistance of strawberry leaves and fruits toward *B. cinerea*. Second, 16S rRNA and ITS2 gene amplicon sequencing was used to study shifts in the bacterial and fungal community in the growing medium (PS or PS + BC) and the strawberry rhizosphere. Third, the nutritional content of the growing medium and strawberry leaves and fruits was evaluated. Finally, RT-qPCR and RNAseq were used to study the changes in the gene expression in strawberry leaves.

### Biochar Induced Resistance to *B. cinerea* in Fruits but Enhanced Susceptibility in Leaves

The effect of adding biochar to the PS on the disease resistance of strawberry was tested. Inoculation assays with *B. cinerea* were performed on both leaves and fruits.

**FIGURE 1:**
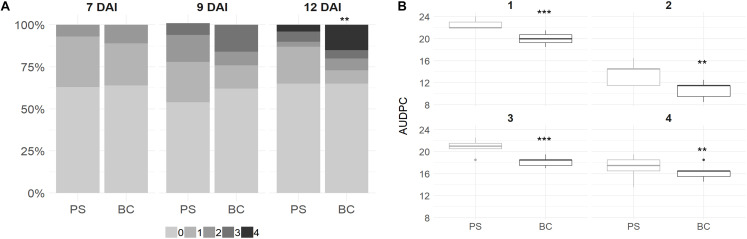
Scoring *Botrytis cinerea* infection on strawberry plant leaves and fruits. **(A)** Strawberry plant leaves were inoculated at 8 weeks of plant growth. In total, three fully expanded leaves per plant were inoculated and per condition [peat substrate (PS)] or biochar-amended PS (PS + BC)] and 12 plants were inoculated. The disease was scored with a value of 0 (no infection) to 4 (100% infected leaf) at 7, 9, and 12 DAI. The score abundances over all plants and leaves are visualized. **(B)** Strawberry fruits (*n* = 6 per time point per tray) were inoculated with *B. cinerea* after picking. Fruits were discarded once infection symptoms were observed, and the AUDPC was calculated based on this measurement and was visualized per treatment. The AUDPC value per tray (*n* = 5) is represented in a boxplot. In total, this was repeated four times, indicated by the values 1–4. Statistical significances are indicated with asterisk (**p < 0.01; ***p < 0.001).

Plant leaves were scored at 7, 9, and 12 DAI with *B. cinerea*. The overall infection rate on plant leaves grown in PS + BC was similar in comparison to the plants grown in PS and even slightly lower at 9 DAI in the biochar-treated plants (Figure 1A). However, when infection occurred on the strawberry leaves grown in PS + BC, the lesion size was larger compared with those grown in PS, indicating a more severe infection 12 days after infection [generalized linear model (GLM), *p* < 0.01; Figure 1A].

In contrast, for strawberry fruits, a decrease in the AUDPC value (LM, *p* < 0.01) was observed for plants grown in PS + BC; this was observed in all four replicates over time (Figure 1B).

In conclusion, the addition of biochar resulted in fewer symptoms on strawberry fruits, while the symptoms on the leaves were more severe.

### Biochar Amendment Leads to Moderate Shifts in the Bacterial and Fungal Community of the Strawberry Rhizosphere

To study if microorganisms increased in and/or were attracted to the plant root, the bacterial and fungal community in the BS and rhizosphere was studied using metabarcoding of the 16 rRNA and ITS2 genes, respectively.

**FIGURE 2:**
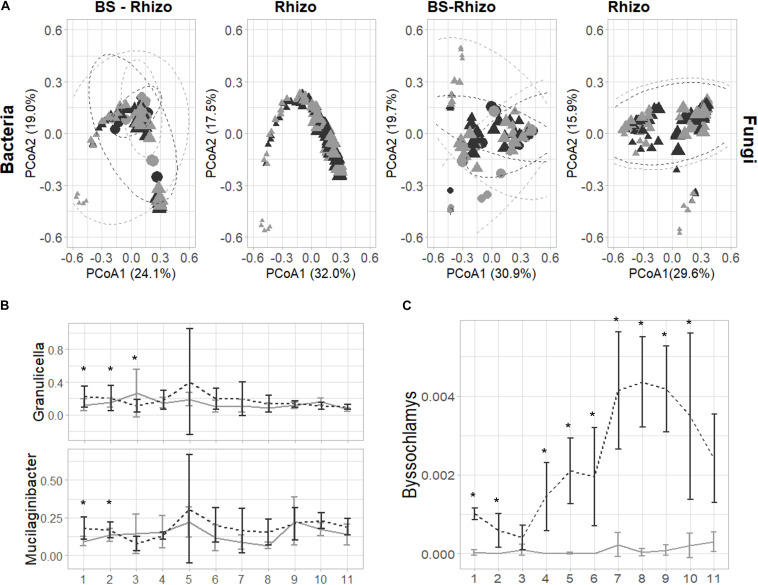
Effect of the addition of biochar on the bacterial (the V3–V4 fragment of the 16S rRNA gene) and fungal (ITS2 gene) community of the strawberry rhizosphere over time. **(A)** The principal coordinate analysis plot showing that the bacterial and fungal community shift over time (symbols are getting bigger when going further in time during the experiment). Only minor effects of the addition of biochar can be observed on the bacterial community (left), while for the fungal community (right), an effect of the addition of biochar can be identified. **(B)** Bacterial genera are consistently increased by the addition of biochar in the first 2–3 weeks of the experiment. **(C)** The fungal genus *Byssochlamys* is consistently increased in the strawberry rhizosphere due to the addition of biochar. For all plots, samples taken from PS are indicated in light gray, while biochar-amended PS is indicated in black. Statistical significances are indicated with an asterisk (*p < 0.05).

After filtering low abundant counts, 3,209 bacterial and 2,073 fungal ASVs were retrieved. No significant differences in bacterial or fungal community diversity were observed in the rhizosphere community, while a trend toward higher fungal diversity in the BS was observed, especially at the start of the experiment (Supplementary Figure 1). The community diversity remained quite stable, but the bacterial and fungal community composition differed between BS and rhizosphere (LM, *p* = 0.001) and varied over time (LM, *p* < 0.001; Supplementary Tables 1, 2 and Figure 2). Furthermore, the addition of biochar to PS shifted the bacterial (PERMANOVA, *p* = 0.001) and fungal (PERMANOVA, *p* = 0.001) community composition. Additionally, the shift induced by the addition of biochar was different when comparing BS and rhizosphere (PERMANOVA, bacteria: *p* = 0.014; fungi: *p* = 0.001) and varied over time (Supplementary Tables 1, 2).

Bacteria and fungi known to influence plant defense can be found in close relation with the plant. Therefore, we first focused on the bacterial and fungal ASVs responsible for these shifts in the strawberry rhizosphere. The PCoA plots and PERMANOVA analyses illustrated that the effect of biochar differed over time: for the bacterial community, biochar only had a noticeable effect in the first 3 weeks. In the first week of the experiment, 24 ASVs increased and 1 decreased in absolute abundance, in the second week, 13 increased and 4 decreased in relative abundance, and in the third week, this diminished to 3 increases and 5 decreases in relative abundance (Supplementary Table 3). Later, the total number of ASVs changed by the addition of biochar, either enriched or depleted, but never exceeded 6. From the 43 ASVs shifted in the first 3 weeks, 5 could be attributed to the genus of *Granulicella* and 10 to the genus *Mucilaginibacter*. When taking all ASVs attributed to either one of these genera into account, both showed a trend toward higher abundance in the strawberry rhizosphere, with a significant change observed only within the first 2 (*Mucilaginibacter*) or 3 (*Granulicella*) weeks (Figure 2B).

The PERMANOVA analysis showed that the effect of biochar was different in BS than in the rhizosphere (Supplementary Figure 2A). The detailed examination revealed that only a limited number of bacterial ASVs changed significantly by the addition of biochar (eight in total, spread over the five time points measured). Most of these ASVs were classified as *Mucilaginibacter* (three) or *Pseudolabrys* (four). In fact, when the effect of biochar on the *Mucilaginibacter* and *Granulicella* genus was analyzed, the same trend is observed as seen in the rhizosphere.

As observed in bacteria, the time of sampling of the fungal community also greatly determined the measured effect of biochar, whereas biochar especially influenced the bacterial community at the start of the experiment, and shifts in the fungal community were almost solely observed from 7 weeks onward (Supplementary Table 4). The taxonomy linked with these ASVs reveals wide diversity. Remarkably, one ASV in the strawberry rhizosphere, classified as *Byssochlamys*, increased consistently (9 out of the 11 weeks) with an increase of 0.001 ± 0.01% to 0.005 ± 0.002% in relative abundance (Figure 2C). However, this genus was almost absent in the BS (Supplementary Figure 2). No other trends can be noticed in the fungal community.

### Biochar Increased the Concentration of Plant-Available N

**FIGURE 3:**
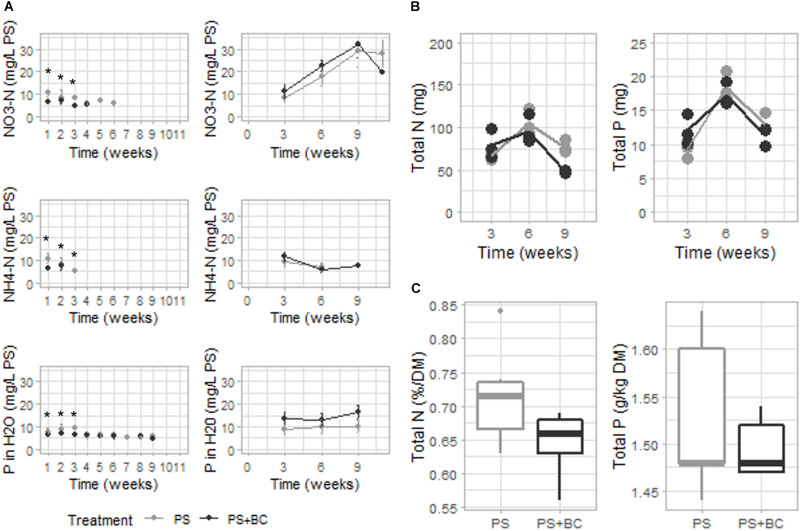
Chemical characterization of the PS with plants, strawberry fresh leaves, and strawberry fruits over 11 weeks of plant growth. **(A)** Plant-available nitrogen (N) (NO_3_-N and NH_4_-N), phosphorous (P) concentrations of either of the PS mixtures [without or with 2 g of biochar per liter added (PS + CH)] in which plants were grown (left), and NO_3_-N, NH_4_-N, and SO_4_ concentrations of either PS mixtures in which no plants were grown (right). Dots represent the mean value with the SD as error bars (*n* = 3). If no values occur, the measurement was below the detection limit. **(B)** Total N and P contents (in mg/pot) measured in plant leaves per plant on weeks 3, 6, and 9 of the experiment. Mean values are represented as lines, and dots represent the values of the biological replicates (*n* = 3). **(C)** Concentrations of N (percentage on DM) and P (in g/kg DM) of strawberry fruits are represented (*n* = 5). Replicates are considered as all fruits picked from all plants grown in either PS or PS + BC at one specific time point (at 5, 6, 7, 8, and 9 weeks of plant growth). Statistical significances are indicated with an asterisk (*p < 0.05).

The nutrient availability of plants was measured each week in the PS in the presence and absence of plants. The concentration of plant-available N (NO_3_-N, NH_4_-N) was significantly lower in biochar-amended PS (GLM, *p* = 0.023) in the first 3 weeks of the experiment, after which the values dropped below the limit of detection. In contrast, in the absence of plants (BS), no effect on either NO_3_-N or NH_4_-N was observed, with even a nonsignificant tendency toward elevated NO_3_-N concentrations for PS + BC (Figure 3A). Furthermore, the addition of biochar led to a significant decrease in P concentration during the first 3 weeks of the experiment (GLM; *p* = 0.009) and led to an increase in SO_4_ concentration in the PS solely in the absence of plants (Supplementary Figures 3, 4). No changes in pH, EC, DM content, and the concentrations of Mg, Ca, K, Na, or C were observed in biochar-amended substrates.

The reduced level of plant-available N and P measured in the growing medium due to the addition of biochar may be due to an increased uptake by the plant. However, when analyzing the N and P concentrations of the strawberry leaves (Figure 3B and Supplementary Table 5) and fruits (Figure 3C and Supplementary Table 6), no statistically significant increase in the concentration could be noted in either plant part. In general, 6 weeks after starting the experiment, the concentrations of N and P in the plant leaves dropped below the optimal range for both plants grown in PS and PS + BC. The addition of biochar resulted in even lower concentrations and the total amount of N in the plant leaves 9 weeks after starting the experiment (Supplementary Table 5). In accordance with the K, Mg, and Ca availability in the PS, biochar amendment did not lead to the changes in the concentrations of K, Mg, or Ca in the strawberry leaves or fruits (Supplementary Tables 5, 6).

This limitation in nutrient availability in the growing medium is not reflected in plant physiological traits. Although the addition of biochar did not change the average plant biomass (both fresh and dry weight [GAM; *p* > 0.05]), an increase in average root weight (GAM; *p* < 0.001) and a nonsignificant tendency toward a bigger strawberry fruit (GLM; *p* = 0.08) were observed (Supplementary Figure 5).

### Addition of Biochar to PS Has a Slightly Negative Effect on the Plant Defense Response in the Leaves

**FIGURE 4:**
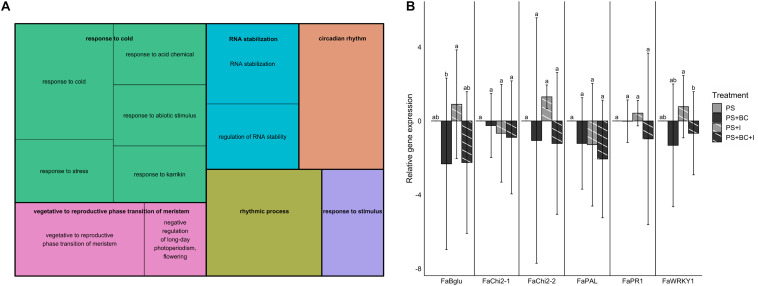
Gene expression in strawberry leaves. **(A)** Gene ontology (GO) enrichment analysis (biological process) of genes differentially expressed in PS vs. PS + BC + I comparison. The size of the boxes corresponds with the significance of the GO terms. **(B)** Relative gene expression values (by reverse transcription quantitative PCR) of six defense genes in strawberry for PS and biochar treatments (PS + BC), inoculated with *B. cinerea* (I) or mock-inoculated. Expression values are expressed in log2 fold changes (mean ± SE). The PS treatment is used as the control treatment; for this treatment, the expression value is set to 0. Statistically significant differences are indicated with different letters.

To test the possible effects of the addition of biochar on the plant defense gene expression in strawberry, leaves sampled 1 week after *B. cinerea* inoculation were used for the RT-qPCR analysis and RNA sequencing. Both inoculated (I) and mock-*B. cinerea* inoculated plants grown in PS or PS + BC were studied to evaluate potential priming of the foliar defense response. Biochar amendment showed no significant effect on the strawberry transcriptome when uninfected. The mere addition of biochar (PS vs. PS + BC) did not appear to upregulate or downregulate any of the genes. Infection of the plants with *B. cinerea* (PS vs. PS + I) resulted in an overall downregulation of host gene expression (86 downregulated, 46 upregulated, *p* = 0.0006313; Supplementary Table 7), as previously noted (De Tender et al., 2021). The addition of biochar (PS vs. PS + BC + I) resulted in a much lower number of upregulated and downregulated genes (14 upregulated genes and 1 downregulated gene, Table 1). To study the biological relevance of these genes, GO enrichment analyses were performed, revealing enrichment of GO terms related to “response to stimulus,” “RNA stability,” and “circadian rhythm” (Figure 4A).

**TABLE 1:** Upregulated and downregulated genes (RNA sequencing) by the addition of biochar and *Botrytis cinerea* infection on the leaves.

Gene ID	Gene annotation	Log2 fold change
FANhyb_rscf00000642.1.g00003.1	Polyphenol oxidase	−8.2
FANhyb_rscf00003700.1.g00001.1	Uncharacterized N-acetyltransferase p20-like	1.3
FANhyb_rscf00001060.1.g00002.1	Chaperone protein DnaJ-like	1.6
FANhyb_rscf00000005.1.g00035.1	Sugar isomerase (SIS) family protein	1.7
FANhyb_rscf00001389.1.g00001.1	Ribonucleoprotein A	1.7
FANhyb_rscf00002652.1.g00001.1	Stress response protein NhaX-like	1.8
FANhyb_rscf00000002.1.g00026.1	DNAJ heat shock N-terminal domain-containing protein	1.9
FANhyb_rscf00000700.1.g00003.1	Hypothetical protein PRUPE_ppa1027140mg	2.2
FANhyb_icon00002769_a.1.g00001.1	Zinc finger protein CONSTANS-LIKE 9-like	2.3
FANhyb_icon00003278_a.1.g00001.1	Hypothetical protein PRUPE_ppa013060mg	2.5
FANhyb_rscf00000382.1.g00011.1	Cold regulated gene 27	2.7
FANhyb_icon19730437_s.1.g00001.1	MATE efflux family protein 6-like	2.8
FANhyb_rscf00000752.1.g00004.1	Unknown protein	3.4
FANhyb_icon00015166_a.1.g00001.1	Auxin responsive SAUR protein	3.4
FANhyb_icon00027985_a.1.g00001.1	Unknown protein	4.1

The RT-qPCR analyses confirmed that there is no induction of defense genes in leaves of biochar-treated plants, either under non-infected (PS + BC vs. PS) or under infected conditions (PS + BC + I vs. PS + I). In accordance with the increased disease response after *B. cinerea* inoculation on strawberry leaves, the addition of biochar even seems to lower the relative gene expression; this was only significant for *Bglu* and *WRKY1* genes (Figure 4B). In general, no evidence for a direct activation (PS + BC) or a primed defense response (PS + BC + I) could be found in biochar-treated vs. control plants.

## Discussion

Biochar amendment in soil and substrate has been shown to have positive effects on plant defense for both soilborne and airborne pathogens (Elad et al., 2010; De Tender et al., 2016b; Frenkel et al., 2017; Jaiswal et al., 2020). Other reports show neutral and even negative effects on plant defense in lettuce and strawberry plants (De Tender et al., 2016a, b). In accordance with those results, our study shows that the addition of biochar resulted in a more severe infection of *B. cinerea* on strawberry leaves, as illustrated by larger lesions. In contrast, infection symptoms on strawberry fruits decreased when biochar was added. This is in accordance with previous findings (De Tender et al., 2016a) where strawberry plants were grown under similar conditions. In contrast, under conditions of strict nutrient limitations, biochar amendment results in positive effects on plant leaves when infected with *B. cinerea* (De Tender et al., 2016b). Such findings point out the importance of the fertilizer dose on the mode of action of biochar. In addition to nutrient availability, previous study has shown that the results are highly dependent on the biochar dose, feedstock, production conditions, and pathosystems (Jaiswal et al., 2020). Amery et al. (2021) illustrated the effect of four types of biochar in strawberry at higher fertilizer application rates. Only one out of four biochar showed a low but significant suppressive effect on *B. cinerea* on the leaves. In studies of the effect of biochar on plant disease, lower concentrations (≤1 wt%) of biochar have been shown to suppress several diseases, while higher concentrations (≥3 wt%) are either largely ineffective or even induce plant disease (Frenkel et al., 2017). For the use as horticultural peat replacement, the recommendation is to standardize biochar feedstocks and concentrations to provide growers with consistent and reproducible biochar with guaranteed effects (Frenkel et al., 2017). For strawberry cultivation, the biggest concern is fruit infection, resulting in yield losses through spoilage. Despite the contradictory effects on the leaves in the current and previous experiments, we can thus conclude that the addition of biochar under low fertilization applications positively affects disease suppression of *B. cinerea* infection on strawberry fruits: all the present experiments show positive results on the fruit, with higher fruit weight noted at the end of the experiment. Standardization of the feedstock, fertilizer dose, and the optimal biochar concentration is thus desirable and should be the focus of future research.

The variability in plant responses and the poor understanding of the mechanisms involved in disease suppression may be one of the key factors hampering the widespread adaptation of biochar as a soil amendment (Jaiswal et al., 2020). The same issues may be found when applying biochar in PS and other growing media. With the aim of generating generally applicable knowledge about biochar and disease suppression, in this study, we elaborated on the effect of biochar on plant defense. We believed that the effect is mediated through (1) changes in the plant nutritional status, (2) changes in the root microbiome, or (3) induction of the plant defense mechanism.

Manipulating the root microbiome through biochar application might be more beneficial in comparison to the use of external biocontrol agents, owing to the difficulties of establishing a stable microbial community and colonizing new species in a complex environment (Herschkovitz et al., 2005). The increase in microbial diversity in and around the plant root has been proposed to facilitate the induction of plant systemic defenses, and previous studies do show higher bacterial diversity under biochar application (De Tender et al., 2016a, b; Kolton et al., 2017). Despite higher abundances of fungal species in the BS, we did not detect increases in bacterial or fungal diversity in the rhizosphere of biochar-amended plants in this study, thus failing to confirm previous results. Another contrast to previous study is that no induction of plant defense genes could be detected in strawberry leaves in the current experiments. There are two possible explanations for this: (1) the fertilizer dose and (2) the applied bioinformatics pipeline. First, in our 2016 experiment (De Tender et al., 2016b), only a low amount of fertilizer was added. In nutrient-limiting situations, biochar proves to be efficient as a nutrient supplier, which can have a direct and positive effect on the diversity of the microbial community and disease resistance. However, in the following report (De Tender et al., 2016a), we also saw a slight increase in diversity, though much less pronounced than in the study by De Tender et al. (2016b). As this study made use of the same experimental setup as in the study by De Tender et al. (2016a), we expected similar results; however, the data analysis procedure was slightly different, as, in this experiment, we analyzed ASVs instead of operational taxonomic units (OTUs). Joos et al. (2020) showed that the use of ASVs instead of OTUs has an immediate effect on the diversity metrics, especially for fungal species. OTUs tend to overestimate the community richness, while ASVs give better results in determining diversity measures.

In addition to the changes in diversity, attracting specific beneficial organisms to the root might help plants cope with pathogens and pests. In previous studies, the addition of biochar has resulted in higher abundances of bacterial families (*Pseudomonadales* and *Flavobacteriales*) and genera (*Bdellovibrio*, *Pseudomonas*, *Rhizobium*, *Rhodanobacter*, and *Trichoderma)* known to contain PGPR, PGPF, and biocontrol organisms (De Tender et al., 2016a, b; Jaiswal et al., 2018, 2019). Negative effects of biochar on beneficial bacteria, such as *Burkholderia ambifaria*, *Pseudomonas chlororaphis*, and *Bacillus pumilus* have also been described (Wu et al., 2020). As observed in the current experiment, the addition of biochar also significantly influenced the root microbiome, with increased abundances of bacteria in the first 3 weeks of plant growth and changes in the fungal community later on in the experiment (from week 7 onward). Interestingly, only three organisms appeared to be consistently influenced, either in the first 3 weeks (*Granulicella*, *Mucilaginibacter*) or over the entire experimental period (*Byssochlamys*). Both *Mucilaginibacter* sp. and *Byssochlamys* sp. have been isolated from the rhizosphere and were shown to include PGPR/PGPF members within their genus (Madhaiyan et al., 2010; Bosso et al., 2016). To verify whether the activity of PGPR and PGPF is, in fact, important for plant defense, we suggested that these organisms should be isolated, cultivated, and tested in disease challenge studies. In addition, an experiment could be setup in which biochar is added to sterilized (e.g., autoclaved) and non-sterilized PS to verify if the effect of biochar on plant defense is regulated through the microbiome solely, or if other biochar-related effects, such as the addition of minerals to the PS, modulate plant physiology and induce resistance in the fruits. The results of this study, in combination with our previous studies on strawberry, show that biochar causes beneficial shifts in the microbial community toward higher abundances of genera related to PGPR/PGPF members. In this study, we focused on the general change in the microbiome, rather than shifts in the specific microorganisms, because these can differ from study to study. Such differences are expected, as each study uses fresh plant material and PS, which are known to be the biggest drivers of the microbial community (Mendes et al., 2013).

The call to look into the longevity of changes in the rhizosphere community after the addition of biochar seems to be valuable (Jaiswal et al., 2019). Specifically, for the bacterial community, the changes in the rhizosphere microbiome only occur within the first week and do not appear to be stable. Three weeks after the start of the experiment, the bacterial community of plants grown in PS or biochar-amended PS showed great similarities. The root microbiome is mainly recruited from the surrounding soil in the early phases of plant development (Garbeva et al., 2004; Bulgarelli et al., 2013). In addition, seedlings seem to be most sensitive to diseases and infection during their very early growth stage (Ben-Yephet and Nelson, 1999; Jaiswal et al., 2019). Modification of the microbiome during the early germination and plant growth can thus provide an additional defense layer.

The fungal genus *Byssochlamys*, which shows great increases in abundance on the addition of biochar, is described as a biocontrol organism producing bioactive compounds (e.g., Rodrigo et al., 2017). The abundance of the genus strongly increases at the time of fruit set. In this study, we noted better disease resistance to *B. cinerea* in the fruit when biochar was present, pointing to a positive effect of the increased prevalence of this genus. Interestingly, this genus is rarely found in the BS, indicating that this organism is specifically related to the strawberry roots.

Compared with peat, biochar contains higher nutrient concentrations, which may affect the nutrient availability in the growing medium (Amery et al., 2021). Previous study has shown that biochar can increase the concentrations of nutrients in PSs. P, K, Ca, and Mg are reported to be present in elevated concentrations, especially under nutrient-limited conditions (De Tender et al., 2016b). However, in this study, the differences in nutrient availability due to the addition of biochar to PS are limited for the pots with plants. Mineral N and water-extractable P, Ca, and Mg concentrations are lower when biochar was added. This may be due to higher plant uptake, sorption onto the biochar, or uptake by the microbial biomass in the growing medium.

Plant susceptibility to pathogens is influenced by the nutritional status of the plant (Nam et al., 2006; Lecompte et al., 2010; Xu et al., 2013). If the observed elevated concentrations of nutrients in the PS observed in this and previous studies also lead to an increased uptake of nutrients by the plant, this could be the basis for the reduced fruit susceptibility to *Botrytis*. For example, higher Ca concentrations increase the plant membrane structural integrity (Bateman and Basham, 1976; Kelman et al., 1989), whereas plants that are deficient in K are less resistant to pests and pathogens (Römheld and Kirkby, 2010). This study shows that despite the significantly lower N concentration in the growing medium in the case of the addition of biochar (indicating elevated uptake), no increased concentrations in either the plant fruits or leaves were noted. In contrast, both the N concentration and the total N uptake in the leaves were lower for the peat amended with biochar. The other nutrients were not altered in the concentration or by the total uptake due to the addition of biochar to the growing medium, with exception of the Mg concentration in the leaves. In comparison with the study of Jaiswal et al. (2018), which shows that nutrient availability to plants is not a factor for plant disease resistance against soilborne pathogens, we can conclude that for our experimental setup, the changes in the nutritional status of the plant are not the main driver regarding plant defense.

To elucidate the effect of the addition of biochar on disease suppression of *B. cinerea* on strawberry (negative for leaves and positive for fruits), we studied if there is an activation or priming of plant defense genes. Initially, we intended to do this on both leaves and fruits, but we were unable to extract a sufficient amount of RNA of good quality from strawberry fruits. Therefore, we chose to work exclusively on the leaves. However, we acknowledged that the study of defense genes and priming by qPCR and RNA sequencing of the fruit is important and valuable, as the most promising results in disease suppression were measured on the fruit. Therefore, we intended to setup new experiments to study defense priming by biochar on strawberry fruits, making use of recently published methods for the extraction of RNA from fruits (Haile et al., 2019).

*Botrytis cinerea* infection leads to general suppression of gene expression in plants grown in the non-amended substrate (De Tender et al., 2021). However, the addition of biochar does also seem to have a negative effect on plant defense in the leaves, with downregulation of several known defense genes in strawberry, two of which were significant (FaBglu, WRKY1). This can explain the more severe infection on the plant leaves. A more in-depth analysis using RNA sequencing shows that biochar has only a minor effect on plant gene expression: only 14 genes are upregulated and 1 downregulated. These results are in contrast with a recent study by Jaiswal et al. (2020) that examined the molecular mechanisms of biochar-elicited suppression of soilborne plant diseases, with a focus on tomato plants. In their experiment, biochar was found to have a priming effect on gene expression of the tomato stem base and upregulated genes involved in plant defense, such as jasmonic acid, brassinosteroids, cytokinins, and auxins. In the study of Meller Harel et al. (2012), an upregulation of defense genes of strawberry leaves is noted when biochar is applied. However, when they applied a dose of 3(w:w)% biochar and inoculated strawberry leaves with *B. cinerea*, the expression of defense genes dropped, even below the expression of the control condition (regular peat, no biochar, and no inoculation), which is in accordance with the observations in this study. Based on these results, we can therefore hypothesize that: (1) the response of the plant is different toward soilborne and airborne pathogens due to the addition of biochar and/or (2) the response depends on the biochar feedstock, application concentration, and the plant system.

Biochar application induced disease resistance on strawberry fruits while increasing the susceptibility toward *B. cinerea* on the leaves. Recently, we observed the opposite effect when chitin is applied to PS: chitin results in a priming response in the strawberry leaves, while the infection on fruit becomes more severe (De Tender et al., 2021). This raises the idea of a possible trade-off between fruits and leaves in terms of defense. Recently, a study on tomatoes has shown that the metabolite composition in leaves and fruits differs among tissues (Nunes-Nesi et al., 2019). More specifically, they show that fruits can contain up to eight times more metabolite quantitative trait loci compared with leaves, indicating that the metabolite levels of leaves are under far greater environmental and probably genetic influence than fruits or seeds. The composition and concentration of plant metabolites can be crucial to mount an effective plant defense response (Ramírez-Gómez et al., 2019). In addition, it has been hypothesized that the changes in the metabolite composition in strawberry fruits lead to changes in disease susceptibility (Wang et al., 2016). Despite the lack of change in the leaf or fruit nutrient content observed in this study, we found differences in the rhizosphere microbiome. Differences in the microbial composition in the rhizosphere are related to changes in the plant metabolite concentration (Jacoby et al., 2021); therefore, the trade-off between fruits and leaves in terms of plant defense might be related to differences in metabolite concentration on the addition of biochar. This could be an interesting focus for future studies.

## Conclusion

The present experiment revealed that in the presence of biochar, a more severe infection with *B. cinerea* was observed on the leaves of strawberry plants, while disease symptoms on fruit were reduced. No effect in nutrient release and uptake by the plant was observed under the addition of biochar, leading us to rule out the role of enhanced nutrient availability in plant defense. Most likely, the disease suppressiveness of biochar-amended peat is mediated by the attraction of biocontrol agents to the plant root, especially in the earliest weeks of plant development. Future experiments should focus on defense gene expression in strawberry fruits, elaborate on the role of the microbiome and disease suppression, and additionally examine possible trade-offs between fruits and leaves in plant defense by studying the metabolites in those tissues.

## Data Availability Statement

The datasets presented in this study can be found in online repositories. The names of the repository/repositories and accession number(s) can be found below: https://www.ncbi.nlm.nih.gov/, PRJNA576171; https://www.ncbi.nlm.nih.gov/, PRJNA576339; https://www.ncbi.nlm.nih.gov/geo/, GSE144526.

## Author Contributions

CD, BVa, TK, and JD performed the conception and/or design of this study. CD and SO collected the data. CD performed the metabarcoding analysis, bioinformatics, statistical data analysis and interpretation, and drafted this study. SO performed the plant physiological data analysis and interpretation. BVa performed the nutrient content analysis and interpretation. BVe performed the RNA sequencing data analysis and interpretation. BVa, BVe, SO, TK, and JD critically revised this study. All authors gave final approval of the version to be published.

## Conflict of Interest

The authors declare that the research was conducted in the absence of any commercial or financial relationships that could be construed as a potential conflict of interest.

## Publisher’s Note

All claims expressed in this article are solely those of the authors and do not necessarily represent those of their affiliated organizations, or those of the publisher, the editors and the reviewers. Any product that may be evaluated in this article, or claim that may be made by its manufacturer, is not guaranteed or endorsed by the publisher.
