# Supplementary material for: Biochar-Enhanced Resistance to Botrytis cinerea in Strawberry Fruits (But Not Leaves) Is Associated With Changes in the Rhizosphere Microbiome
Source: Front Plant Sci. 2021 Aug 23;12:700479. doi: 10.3389/fpls.2021.700479 (PMC8419269; doi:10.3389/fpls.2021.700479)
Supplement: Supplementary file 1 [file Data_Sheet_1.docx]

***Supplementary Material***

Biochar-enhanced resistance to *Botrytis cinerea* in strawberry fruits (but not leaves) is associated with changes in the rhizosphere microbiome

Caroline De Tender^1,2^, Bart Vandecasteele^1^, Bruno Verstraeten^3^, Sarah Ommeslag^1^, Tina Kyndt^3^, Jane Debode^1^


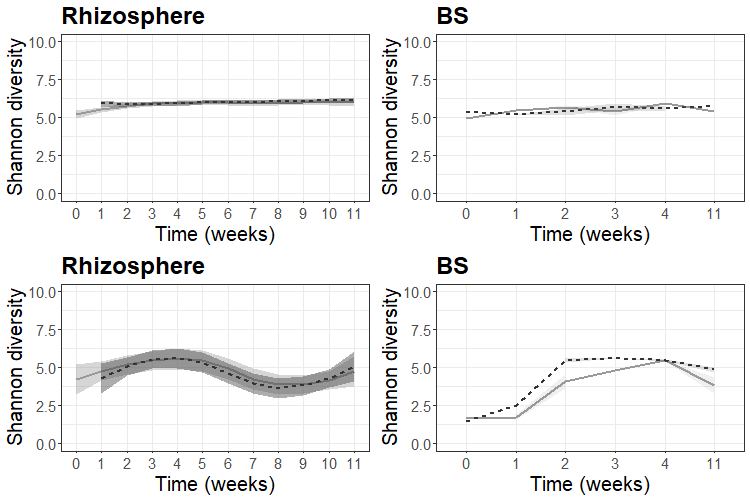


**Figure S1 Visualization diversity (Shannon index) of the bacterial (top) and fungal (down) community of the rhizosphere (left) and bulk peat substrate (BS; right**). Values represent mean values (n= 5 for rhizosphere and n=3 for bulk growing medium) with standard error illustrated as a shaded zone surrounding the mean. Solid gray line = samples from non-amended peat substrate, black dashed line = 2 g/L biochar amended peat substrate.


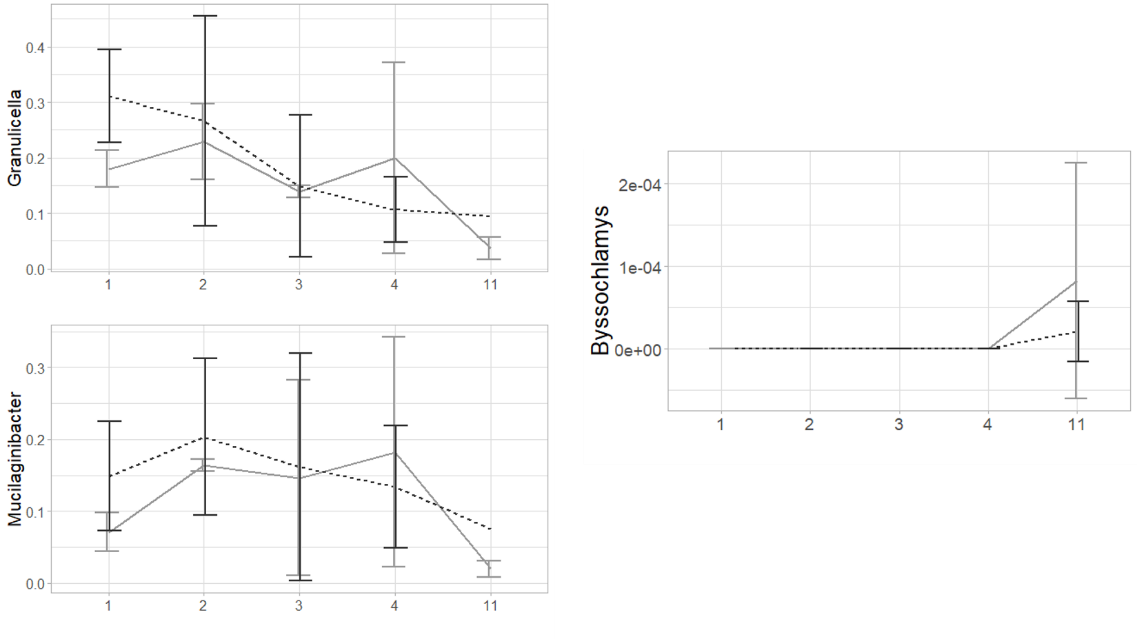


**Figure S2: Effect of biochar addition on the bacterial (V3-V4 fragment of the 16S rRNA gene) and fungal (ITS2 gene) community of the bulk peat substrate (BS) and peat substrate (PS) over time**. Bacterial and fungal genera that showed consistent increases under biochar addition in the rhizosphere are shown. For bacteria, the same trend is observed, whereas the fungal genus *Byssochlamys* is almost absent in BS. For all plots, samples taken from peat substrate (PS) are indicated in light gray, while biochar-amended PS is indicated in black.


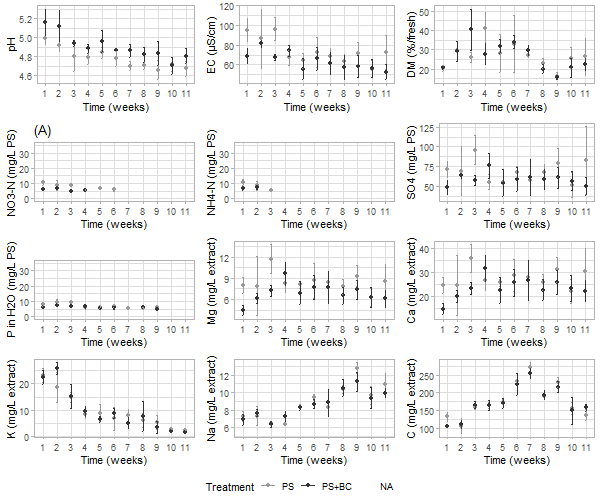


**Figure S3: EC (µS/cm), pH (-) and water-extractable elements (mg/L peat substrate (PS)) for the peat substrate (pots with plants) over 11 weeks of plant growth.** Dots represent the mean value with the standard deviation as error bars (n=3). If no values occur, the measurement was below detection limit (EC: Electrical conductivity; DM: Dry Matter).


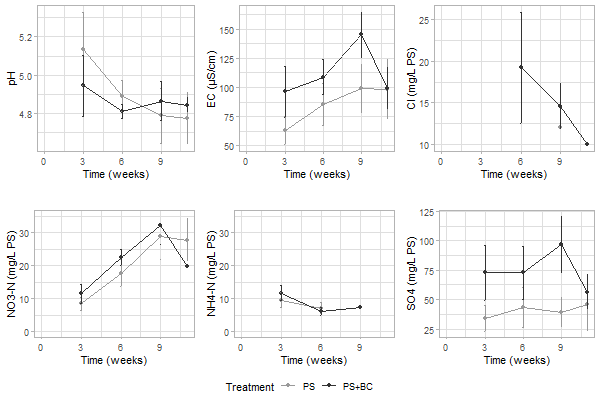


**Figure S4:** **EC (µS/cm), pH (-) and water-extractable elements (mg/L peat substrate (PS)) for the peat substrate (pots without plants) over 11 weeks of plant growth.** Measures were taken on week 3, 6, 9 and 11 of the experiment. Dots represent the mean value with the standard deviation as error bars (n=3). If no values occur, the measurement was below detection limit (EC: Electrical conductivity).


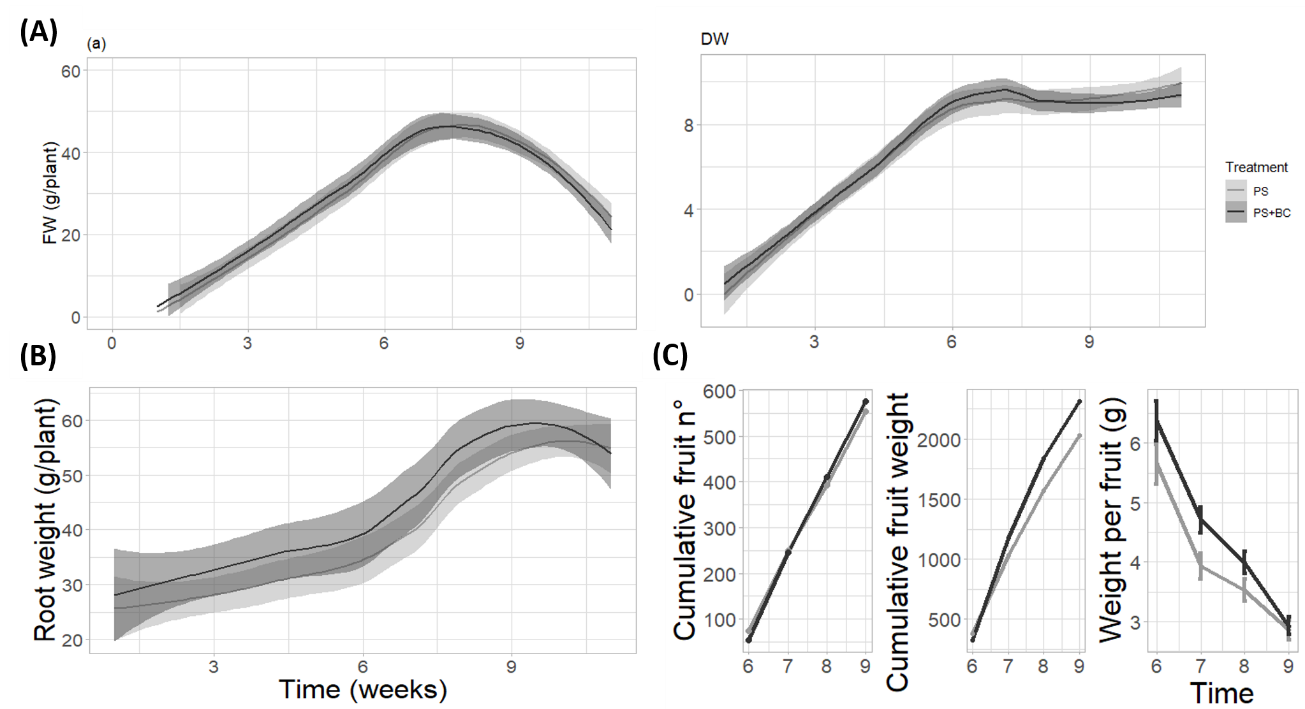


**Figure S5 Changes in plant physiological parameters by the addition of biochar to PS.** **(A)** Fresh weight and dry weight of the strawberry plant by biochar addition. No significant changes were noted (n=6). **(B)** Root weight is positively influenced by the addition of biochar to PS. **(C)** Effect of biochar on strawberry fruit. Biochar had no effect on the fruit number (left) a positive effect on total fruit weight (middle) , with an overall increase of fruit weight and a higher weight per fruit (right). For all figures, PS is indicated in gray, while PS+BC is indicated in black.

**Table S1: Statistical significance of Permutational Analysis of Variance (PERMANOVA) test on the ASV table of the bacterial community.** Variables include Treatment (no biochar versus biochar addition), Time (weekly measurement from the start of the experiment (=0) to the end of the experiment (=11 weeks)) and sample type (bulk growing medium (BS) and rhizosphere). Statistically significant results are illustrated by either *p < 0.05; **p < 0.01 or ***p<0.001.

Call:

adonis(formula = dissimilarity_matrix_PS_CH ~ Treatment * Time * SampleType)

Permutation: free

Number of permutations: 999

Terms added sequentially (first to last)

Df SumsOfSqs MeanSqs F.Model R2 Pr(>F)

Treatment 1 0.3489 0.34887 4.7221 0.01279 0.001 ***

Time 11 12.1126 1.10114 14.9044 0.44401 0.001 ***

SampleType 3 2.4002 0.80008 10.8294 0.08799 0.001 ***

Treatment:Time 11 1.5780 0.14346 1.9417 0.05785 0.001 ***

Treatment:SampleType 1 0.1869 0.18694 2.5304 0.00685 0.014 *

Time:SampleType 5 2.5042 0.50084 6.7790 0.09180 0.001 ***

Treatment:Time:SampleType 4 0.3916 0.09791 1.3252 0.01436 0.111

Residuals 105 7.7574 0.07388 0.28436

Total 141 27.2799 1.00000

---

**Table S2: Statistical significance of Permutational Analysis of Variance (PERMANOVA) test on the ASV table of the fungal community.** Variables include Treatment (no biochar versus biochar addition), Time (weekly measurement from the start of the experiment (=0) to the end of the experiment (=11 weeks)) and sample type (bulk growing medium (BS) and rhizosphere). Statistically significant results are illustrated by either *p < 0.05; **p < 0.01 or ***p<0.001.

Call:

adonis(formula = dissimilarity_matrix_PS_CHF ~ TreatmentF * TimeF * SampleTypeF)

Permutation: free

Number of permutations: 999

Terms added sequentially (first to last)

Df SumsOfSqs MeanSqs F.Model R2 Pr(>F)

TreatmentF 1 0.309 0.30913 4.2244 0.00932 0.007 **

TimeF 11 18.561 1.68738 23.0588 0.55984 0.001 ***

SampleTypeF 3 1.763 0.58754 8.0289 0.05316 0.001 ***

TreatmentF:TimeF 11 1.948 0.17705 2.4194 0.05874 0.001 ***

TreatmentF:SampleTypeF 1 0.475 0.47454 6.4847 0.01431 0.001 ***

TimeF:SampleTypeF 5 1.828 0.36563 4.9964 0.05514 0.001 ***

TreatmentF:TimeF:SampleTypeF 4 0.441 0.11028 1.5070 0.01330 0.069 .

Residuals 107 7.830 0.07318 0.23617

Total 143 33.154 1.00000

---

Signif. codes: 0 ‘***’ 0.001 ‘**’ 0.01 ‘*’ 0.05 ‘.’ 0.1 ‘ ’ 1

**Table S3** **Enrichments and depletions of bacterial ASVs with biochar treatment.** Biochar-induced increase in relative abundance at a specific time point (week 1 (t1) to week 11 (t11)), is indicated by +. Statistically significant (p<0.05) decreases in relative abundance are indicated by – .

| **ASV** | **Genus** | t1 | t2 | t3 |
| --- | --- | --- | --- | --- |
| 107 | Acidocella |  |  | - |
| 796 | Amnibacterium | + |  |  |
| 265 | Arsenicitalea |  | - |  |
| 63 | Bryobacter | + | + |  |
| 188 | Candidatus_Koribacter | + |  |  |
| 477 | Candidatus_Ovatusbacter |  | + |  |
| 548 | Caulobacter |  | - |  |
| 118 | Devosia | + |  |  |
| 37 | Granulicella |  | + |  |
| 167 | Granulicella | + | + | + |
| 233 | Granulicella |  | + |  |
| 290 | Granulicella | + |  |  |
| 390 | Granulicella | + |  |  |
| 208 | Haliangium | + |  |  |
| 544 | Haliangium | + |  |  |
| 602 | Iamia | + | + |  |
| 2169 | Lysobacter |  |  | + |
| 162 | Methylovirgula | + |  |  |
| 68 | Mucilaginibacter | + |  |  |
| 78 | Mucilaginibacter | + |  |  |
| 159 | Mucilaginibacter | + |  |  |
| 181 | Mucilaginibacter | + |  |  |
| 301 | Mucilaginibacter |  | + |  |
| 365 | Mucilaginibacter | + |  |  |
| 380 | Mucilaginibacter |  | + |  |
| 426 | Mucilaginibacter |  | + |  |
| 431 | Mucilaginibacter |  | + |  |
| 926 | Mucilaginibacter | + |  |  |
| 54 | Occallatibacter | + |  |  |
| 119 | Occallatibacter | + |  |  |
| 253 | Occallatibacter | + |  |  |
| 295 | Occallatibacter |  | + |  |
| 505 | Occallatibacter | + |  |  |
| 21 | Opitutus |  |  | - |
| 80 | Opitutus | + |  |  |
| 2045 | Rahnella |  | + |  |
| 425 | Reyranella |  |  | + |
| 859 | Rhodanobacter |  |  | - |
| 349 | Roseiarcus | + | + |  |
| 327 | Schlesneria |  | - |  |
| 807 | Spirochaeta_2 | - |  |  |
| 2233 | SWB02 |  | - |  |
| 359 | Terracidiphilus | + |  | - |

**Table S4** **Enrichments and depletions of fungal ASVs with biochar treatment.** Biochar-induced increase in relative abundance at a specific time point (week 1 (t1) to week 11 (t11)), is indicated by +. Statistically significant (p<0.05) decreases in relative abundance are indicated by – .

| **ASV** | **Genus** | t1 | t2 | t3 | t4 | t5 | t6 | t7 | t8 | t9 | t10 | t11 |
| --- | --- | --- | --- | --- | --- | --- | --- | --- | --- | --- | --- | --- |
| 3 | *Basidiodendron* |  |  |  |  |  |  | - |  |  |  |  |
| 14 | *Mortierella* |  |  |  |  |  |  |  |  | + |  |  |
| 26 | *Fusarium* |  |  |  |  |  |  |  |  | + |  |  |
| 28 | *Clonostachys* |  |  |  |  |  |  |  |  |  | - |  |
| 34 | *Clavaria* |  |  |  |  |  |  | - | - |  |  |  |
| 41 | *Trichoderma* |  |  |  |  |  |  |  |  | - |  |  |
| 45 | *Plectosphaerella* |  |  |  |  |  |  |  |  |  | - |  |
| 47 | *Cladosporium* |  |  |  |  |  |  | + |  |  |  |  |
| 104 | *Podospora* |  |  |  |  |  |  |  |  | + |  |  |
| 105 | *Delicatula* |  |  |  |  |  |  |  | + | + | + |  |
| 124 | *Oidiodendron* |  |  |  |  |  |  |  |  |  |  | - |
| 130 | *Stachybotrys* |  |  |  |  |  |  |  |  | + |  |  |
| 132 | *Acidomelania* |  |  |  |  |  |  |  | - |  |  | + |
| 140 | *Archaeorhizomyces* | |  |  |  |  |  | - |  | + |  |  |
| 146 | *Subulicystidium* |  |  |  |  |  |  |  |  | + |  |  |
| 153 | *Clavispora* |  |  |  |  |  |  | + |  |  |  | + |
| 155 | *Microscypha* |  |  |  |  |  |  | + | + |  |  |  |
| 163 | *Microdochium* | + |  |  |  |  |  |  |  | + |  |  |
| 175 | *Neobulgaria* |  |  |  |  |  |  |  | - |  |  |  |
| 178 | *Calyptrozyma* |  |  |  |  |  |  |  |  | + |  |  |
| 183 | *Sarcoleotia* |  |  |  |  |  |  |  |  |  | - | + |
| 187 | *Rhizophagus* |  |  |  |  |  |  | + |  |  |  |  |
| 191 | *Lachnum* |  |  |  |  |  |  |  |  | + |  | + |
| 193 | *Rhexocercosporidium* | |  |  |  |  |  |  |  |  | - |  |
| 199 | *Tetrachaetum* |  |  |  |  |  |  | - |  |  |  |  |
| 204 | *Gibellulopsis* |  |  |  |  |  |  | + | - | + |  |  |
| 205 | *Holtermanniella* |  |  |  |  |  |  |  | + |  |  |  |
| 212 | *Boothiomyces* |  |  |  |  |  |  |  | - | + |  |  |
| 224 | *Geoglossum* |  |  |  |  |  |  |  |  | + |  |  |
| 258 | *Zopfiella* |  |  |  |  |  |  |  | + |  |  |  |
| 259 | *Guehomyces* |  |  |  |  |  |  | + |  |  |  |  |
| 274 | *Alternaria* |  |  |  |  |  |  |  | + |  |  |  |
| 289 | *Ophiosphaerella* |  |  |  |  |  |  |  | - | + | - |  |
| 298 | *Claroideoglomus* | - |  |  |  |  |  | - |  |  |  |  |
| 305 | *Cercophora* |  |  |  |  |  |  | + |  |  | - |  |
| 307 | *Pseudaleuria* |  |  |  |  |  |  |  |  | + |  |  |
| 312 | *Pyrenochaetopsis* | |  |  |  |  |  | - |  | + | + |  |
| 317 | *Peniophora* |  |  |  |  |  |  |  |  | + | - |  |
| 318 | *Gaeumannomyces* | |  |  |  |  |  |  | + |  |  | - |
| 325 | *Dimorphospora* |  |  |  |  |  |  |  |  | - |  |  |
| 336 | *Coprinus* |  |  |  |  |  |  |  |  | + |  |  |
| 337 | *Trebouxia* |  |  |  |  |  |  |  | - | + |  |  |
| 339 | *Plenodomus* |  |  |  |  |  |  | + | - |  |  |  |
| 341 | *Wallemia* |  |  |  |  |  |  | + |  |  |  |  |
| 355 | *Codinaea* |  |  |  |  |  |  |  |  | + |  |  |
| 382 | *Byssochlamys* | + | + |  | + | + | + | + | + | + | + |  |
| 384 | *Eocronartium* |  |  |  |  |  |  |  |  |  | - |  |
| 391 | *Trichocladium* |  |  |  |  |  |  |  |  |  | - |  |
| 395 | *Cladorrhinum* |  |  |  |  |  |  |  | - | + | - |  |
| 399 | *Apodospora* |  |  |  |  |  |  |  | + | + |  |  |
| 408 | *Sistotrema* |  |  |  |  |  |  |  |  | - |  |  |
| 460 | *Hygrocybe* |  |  |  |  |  |  |  | + | + |  |  |
| 477 | *Chloridium* |  |  |  |  |  |  | + |  |  |  |  |
| 479 | *Periconia* |  |  |  |  |  |  |  | - |  | + |  |
| 498 | *Slopeiomyces* |  |  |  |  |  |  |  | + | + |  |  |
| 505 | *Leucosporidium* |  |  |  |  |  | + |  |  |  |  |  |
| 541 | *Naganishia* |  |  |  |  |  |  | + |  |  |  |  |
| 553 | *Clitopilus* |  |  |  |  |  |  |  |  |  | - | - |
| 627 | *Drechslera* |  |  |  |  |  |  |  |  | + | = |  |
| 657 | *Blastobotrys* |  |  | + |  |  |  |  |  |  |  |  |
| 660 | *Leptosphaeria* |  |  |  |  |  |  |  |  | + |  |  |
| 666 | *Entorrhiza* |  |  |  |  |  |  |  | - | = |  |  |
| 688 | *Lindgomyces* |  |  |  |  |  |  |  |  | + | - |  |
| 708 | *Nidulariopsis* |  |  |  |  |  |  |  | + | + |  |  |
| 720 | *Paraphoma* |  |  |  |  |  |  |  |  |  | - |  |
| 739 | *Hannaella* |  |  |  |  |  |  |  |  |  |  | + |
| 748 | *Ophiocordyceps* |  |  |  |  |  |  |  | + | + |  |  |
| 768 | *Fusicolla* |  |  |  |  |  |  |  | - |  |  |  |
| 790 | *Lachnella* |  |  |  |  |  |  |  |  | + |  |  |
| 812 | *Scytalidium* |  |  |  |  |  | + |  |  |  |  |  |
| 857 | *Henningsomyces* |  |  |  |  |  |  | - |  |  |  |  |
| 1011 | *Lecythophora* |  |  |  |  |  |  |  |  | + |  |  |
| 1031 | *Torula* |  |  |  |  |  |  |  |  |  | - |  |
| 1099 | *Arthrinium* |  |  |  |  |  |  |  |  |  | - |  |
| 1110 | *Cistella* |  |  |  |  |  |  | - |  |  |  | + |
| 1119 | *Psilocybe* |  |  |  |  |  |  |  |  | + |  |  |
| 1121 | *Cyphellophora* |  |  |  |  |  |  |  |  |  | + |  |
| 1177 | *Pulvinula* |  |  |  |  |  |  | + |  |  |  |  |
| 1224 | *Leohumicola* |  |  |  |  |  |  |  |  | + |  |  |
| 1277 | *Glomus* |  |  |  |  |  |  |  |  |  | - | + |
| 1359 | *Basidiobolus* |  |  |  |  |  |  |  |  | + |  |  |

**Table S5:** **Nutrient concentrations (a) and total uptake (b) in strawberry leaves after 3, 6 and 9 weeks of strawberry plant growth in the time course experiment.** Values are represented as mean values ± standard deviation (n=3). Values for concentration are indicated as %/DM (N), g/kg dry matter (P, K, Mg, Ca) or mg/kg dry matter (Fe, Mn). Values for total uptake are indicated in mg.

|  |  | **3 weeks** | | **6 weeks** | | | **9 weeks** | | |
| --- | --- | --- | --- | --- | --- | --- | --- | --- | --- |
| **(a)** | **Optimal range** | **PS** | **PS+BC** | | **PS** | **PS+BC** | | **PS** | **PS+BC** |
| **N** | 2.0-2.8 | 2.6±0.1 (c) | 2.6±0.1 (c) | | 1.1±0.1 (b) | 1.1±0.1 (b) | | 1.0±0.1 (b) | 0.6±0.1 (a) |
| **P** | 2.5-4.0 | 3.7±0.1 (c) | 4.0±0.1 (c) | | 2.0±0.1 (b) | 1.9±0.1 (b) | | 1.6±0.1 (a) | 1.5±0.1 (a) |
| **K** | 15-25 | 19.2±1.0 (c) | 20.9±0.8 (c) | | 8.8±2.1 (b) | 8.3±0.4 (b) | | 4.2±0.8 (a) | 4.9±0.9 (a) |
| **Mg** | 3.0-5.0 | 4.6±0.1 (b) | 4.3±0.2 (b) | | 3.5±0.1 (a) | 3.2±0.1 (a) | | 4.4±0.1 (b) | 3.6±0.2 (a) |
| **Ca** | 7.0-17 | 8..1±0.1 (a) | 8.0±0.4 (a) | | 7.5±0.7 (a) | 7.5±0.1 (a) | | 10.4±0.4 (b) | 9.8±05 (b) |
| **(b)** |  |  |  | |  |  | |  |  |
| **N** |  | 66±4.3 (ab) | 79.4±16.4 (bc) | | 105±14.5 (c) | 95.8±16.6 (c) | | 77±7.4 (b) | 47.9±1.9 (a) |
| **P** |  | 9.4±1.3 (a) | 12.0±2.3 (a) | | 18.5±2.1 (b) | 17.2±1.7 (b) | | 12.9±1.4 (a) | 11.4±1.4 (a) |
| **K** |  | 48.9±6.4 (ab) | 63.0±11.3 (b) | | 81.5±26.0 (b) | 74.9±3.1 (b) | | 34.7±8.9 (a) | 37.9±7.5 (a) |
| **Mg** |  | 11.5±1.4 (a) | 12.8±2.2 (a) | | 31.7±3.5 (b) | 28.8±2.9 (b) | | 35.4±2.9 (b) | 27.4±2.7 (b) |
| **Ca** |  | 20.7±3.0 (a) | 24.0±4.0 (a) | | 69.3±10.3 (b) | 67.3±5.8 (b) | | 85.5±8.0 (b) | 75.1±5.4 (b) |

**Table S6: Nutrient concentrations (a) and total uptake (b) in strawberry fruit.** Values are represented as mean values ± standard deviation (n=3). (a) Values for concentration are indicated as %/DM (N), g/kg dry matter (P, K, Mg, Ca). (b) Values for total uptake are indicated in mg and could only be calculated for fruits taken 7 weeks after starting the experiment.

|  | | **PS** | **PS+BC** |
| --- | --- | --- | --- |
| **(a)** | |  |  |
| **N** | 0.71±0.07 (a) | | 0.64±0.05 (a) |
| **P** | | 1.51±0.09 (a) | 1.50±0.03 (a) |
| **K** | | 8.41±0.40 (a) | 8.82±0.39 (a) |
| **Mg** | | 1.54±0.33 (a) | 1.48±0.22 (a) |
| **Ca** | | 2.74±0.68 (a) | 2.58±0.45 (a) |
| **(b)** | |  |  |
| **N** | | 45 | 45.7 |
| **P** | | 9.5 | 10.6 |
| **K** | | 52.3 | 63.5 |
| **Mg** | | 9.6 | 10.2 |
| **Ca** | | 17.1 | 18.4 |

**Table S7: Up- and downregulated genes (RNA sequencing) by B. cinerea infection on the leaves (PS vs. PS+I).**

| **Gene ID** | **logFC** |
| --- | --- |
| FANhyb_icon00000056_a.1.g00002.1 | -1.46328 |
| FANhyb_icon00000286_a.1.g00001.1 | -1.65077 |
| FANhyb_icon00000491_a.1.g00001.1 | -1.59752 |
| FANhyb_icon00000519_a.1.g00001.1 | 2.623343 |
| FANhyb_icon00000804_a.1.g00001.1 | 1.205036 |
| FANhyb_icon00001250_a.1.g00001.1 | 2.04092 |
| FANhyb_icon00001351_a.1.g00001.1 | -3.48359 |
| FANhyb_icon00001395_a.1.g00001.1 | -2.37085 |
| FANhyb_icon00001624_a.1.g00001.1 | -1.81845 |
| FANhyb_icon00002769_a.1.g00001.1 | 2.092776 |
| FANhyb_icon00003078_a.1.g00001.1 | -1.41588 |
| FANhyb_icon00003278_a.1.g00001.1 | 2.988231 |
| FANhyb_icon00003811_a.1.g00001.1 | -1.82469 |
| FANhyb_icon00006080_a.1.g00001.1 | 1.812699 |
| FANhyb_icon00006480_a.1.g00001.1 | -1.53254 |
| FANhyb_icon00006642_a.1.g00001.1 | -1.7653 |
| FANhyb_icon00007591_a.1.g00001.1 | -2.00512 |
| FANhyb_icon00007641_a.1.g00001.1 | 2.769313 |
| FANhyb_icon00008755_a.1.g00001.1 | 3.663744 |
| FANhyb_icon00008971_a.1.g00001.1 | -2.41087 |
| FANhyb_icon00009318_a.1.g00001.1 | -2.02074 |
| FANhyb_icon00010779_a.1.g00001.1 | -1.56057 |
| FANhyb_icon00011188_a.1.g00001.1 | -3.18512 |
| FANhyb_icon00015158_a.1.g00001.1 | 5.86393 |
| FANhyb_icon00016429_a.1.g00001.1 | -2.43218 |
| FANhyb_icon00018851_a.1.g00001.1 | -2.16913 |
| FANhyb_icon00020670_a.1.g00001.1 | -2.36458 |
| FANhyb_icon00027359_a.1.g00001.1 | -3.83043 |
| FANhyb_icon00030108_a.1.g00001.1 | -2.82337 |
| FANhyb_icon00030158_a.1.g00001.1 | -2.14398 |
| FANhyb_icon00030879_a.1.g00001.1 | -2.06324 |
| FANhyb_icon00031686_a.1.g00001.1 | -3.08189 |
| FANhyb_icon00035972_a.1.g00001.1 | -4.31642 |
| FANhyb_icon00038336_a.1.g00001.1 | -2.38021 |
| FANhyb_icon00039674_a.1.g00001.1 | 1.947749 |
| FANhyb_icon00040024_a.1.g00001.1 | -6.38869 |
| FANhyb_icon00044428_a.1.g00001.1 | 1.454863 |
| FANhyb_icon00045239_a.1.g00001.1 | -2.1469 |
| FANhyb_icon19625041_s.1.g00001.1 | -3.11226 |
| FANhyb_icon19730437_s.1.g00001.1 | 4.068712 |
| FANhyb_icon20235835_s.1.g00001.1 | -3.5418 |
| FANhyb_rscf00000002.1.g00026.1 | 2.223493 |
| FANhyb_rscf00000003.1.g00046.1 | -4.26686 |
| FANhyb_rscf00000005.1.g00035.1 | 1.765768 |
| FANhyb_rscf00000008.1.g00049.1 | 1.992491 |
| FANhyb_rscf00000012.1.g00021.1 | 3.770001 |
| FANhyb_rscf00000013.1.g00025.1 | -2.14136 |
| FANhyb_rscf00000015.1.g00031.1 | -3.01656 |
| FANhyb_rscf00000024.1.g00007.1 | 1.096966 |
| FANhyb_rscf00000030.1.g00022.1 | -3.58355 |
| FANhyb_rscf00000036.1.g00021.1 | 3.640636 |
| FANhyb_rscf00000040.1.g00016.1 | -1.55709 |
| FANhyb_rscf00000046.1.g00002.1 | -1.91905 |
| FANhyb_rscf00000046.1.g00011.1 | -1.09269 |
| FANhyb_rscf00000046.1.g00026.1 | -1.64534 |
| FANhyb_rscf00000047.1.g00005.1 | 1.402856 |
| FANhyb_rscf00000049.1.g00022.1 | -1.32629 |
| FANhyb_rscf00000050.1.g00021.1 | 3.952462 |
| FANhyb_rscf00000078.1.g00008.1 | -1.96526 |
| FANhyb_rscf00000087.1.g00005.1 | 1.417515 |
| FANhyb_rscf00000112.1.g00005.1 | 1.937623 |
| FANhyb_rscf00000129.1.g00011.1 | -2.28517 |
| FANhyb_rscf00000141.1.g00004.1 | 1.36689 |
| FANhyb_rscf00000160.1.g00010.1 | -1.0189 |
| FANhyb_rscf00000160.1.g00014.1 | 1.366491 |
| FANhyb_rscf00000177.1.g00014.1 | 1.060159 |
| FANhyb_rscf00000187.1.g00018.1 | -1.50299 |
| FANhyb_rscf00000201.1.g00002.1 | -2.41701 |
| FANhyb_rscf00000219.1.g00018.1 | 1.840994 |
| FANhyb_rscf00000223.1.g00009.1 | -2.14999 |
| FANhyb_rscf00000235.1.g00009.1 | -1.7566 |
| FANhyb_rscf00000272.1.g00003.1 | -1.93362 |
| FANhyb_rscf00000295.1.g00001.1 | -2.2763 |
| FANhyb_rscf00000328.1.g00001.1 | 2.427299 |
| FANhyb_rscf00000354.1.g00005.1 | -1.21667 |
| FANhyb_rscf00000359.1.g00002.1 | -2.78798 |
| FANhyb_rscf00000382.1.g00011.1 | 3.018209 |
| FANhyb_rscf00000425.1.g00006.1 | -2.54808 |
| FANhyb_rscf00000476.1.g00003.1 | 4.944425 |
| FANhyb_rscf00000495.1.g00008.1 | -3.35668 |
| FANhyb_rscf00000515.1.g00008.1 | 1.265377 |
| FANhyb_rscf00000545.1.g00004.1 | -2.56291 |
| FANhyb_rscf00000569.1.g00007.1 | -3.68875 |
| FANhyb_rscf00000637.1.g00004.1 | -2.20429 |
| FANhyb_rscf00000647.1.g00001.1 | -2.21105 |
| FANhyb_rscf00000660.1.g00009.1 | -3.09054 |
| FANhyb_rscf00000699.1.g00002.1 | 1.231724 |
| FANhyb_rscf00000702.1.g00005.1 | -1.93811 |
| FANhyb_rscf00000752.1.g00004.1 | 4.299374 |
| FANhyb_rscf00000773.1.g00007.1 | -2.22147 |
| FANhyb_rscf00000787.1.g00001.1 | 1.31778 |
| FANhyb_rscf00000804.1.g00001.1 | 1.985126 |
| FANhyb_rscf00000891.1.g00002.1 | -1.79605 |
| FANhyb_rscf00000923.1.g00002.1 | -2.19737 |
| FANhyb_rscf00000957.1.g00002.1 | -2.15449 |
| FANhyb_rscf00000991.1.g00008.1 | -2.51299 |
| FANhyb_rscf00001060.1.g00002.1 | 1.804379 |
| FANhyb_rscf00001201.1.g00004.1 | -1.20825 |
| FANhyb_rscf00001213.1.g00003.1 | -1.88722 |
| FANhyb_rscf00001242.1.g00002.1 | 1.991122 |
| FANhyb_rscf00001253.1.g00003.1 | -1.98118 |
| FANhyb_rscf00001329.1.g00002.1 | -1.51067 |
| FANhyb_rscf00001389.1.g00001.1 | 2.439888 |
| FANhyb_rscf00001408.1.g00001.1 | -3.41108 |
| FANhyb_rscf00001605.1.g00002.1 | -1.65177 |
| FANhyb_rscf00001608.1.g00001.1 | -1.49103 |
| FANhyb_rscf00001917.1.g00001.1 | -2.09968 |
| FANhyb_rscf00002071.1.g00001.1 | 1.245997 |
| FANhyb_rscf00002141.1.g00001.1 | -1.69813 |
| FANhyb_rscf00002433.1.g00001.1 | -1.95202 |
| FANhyb_rscf00002447.1.g00002.1 | -4.45129 |
| FANhyb_rscf00002550.1.g00001.1 | 2.306307 |
| FANhyb_rscf00002652.1.g00001.1 | 1.871111 |
| FANhyb_rscf00002735.1.g00001.1 | -2.17595 |
| FANhyb_rscf00002799.1.g00001.1 | -2.57553 |
| FANhyb_rscf00003077.1.g00001.1 | -2.04258 |
| FANhyb_rscf00003576.1.g00001.1 | -2.25355 |
| FANhyb_rscf00003837.1.g00001.1 | -2.39717 |
| FANhyb_rscf00003859.1.g00001.1 | -4.21937 |
| FANhyb_rscf00004064.1.g00001.1 | -1.42268 |
| FANhyb_rscf00004717.1.g00002.1 | 2.092764 |
| FANhyb_rscf00004895.1.g00001.1 | -1.92429 |
| FANhyb_rscf00004952.1.g00001.1 | -1.41259 |
| FANhyb_rscf00005149.1.g00001.1 | 4.646209 |
| FANhyb_rscf00005343.1.g00002.1 | -1.69946 |
| FANhyb_rscf00005491.1.g00001.1 | 2.17172 |
| FANhyb_rscf00005522.1.g00001.1 | 2.82753 |
| FANhyb_rscf00005614.1.g00001.1 | -3.46321 |
| FANhyb_rscf00005726.1.g00001.1 | -7.27607 |
| FANhyb_rscf00006543.1.g00001.1 | 1.736176 |
| FANhyb_rscf00006551.1.g00001.1 | 3.024366 |
| FANhyb_rscf00007376.1.g00001.1 | -3.57314 |
| FANhyb_rscf00007376.1.g00001.1 -3.573140 |  |
